# Design of dual-band power amplifier using bandstop filter and dual-mode bias circuit for multistandard transceiver systems

**DOI:** 10.1038/s41598-023-42821-8

**Published:** 2023-09-22

**Authors:** Sepehr Zarghami, Mohsen Hayati

**Affiliations:** Electrical Engineering Department, Faculty of Engineering, Razi University, Kermanshah, 67149-67346 Iran

**Keywords:** Electrical and electronic engineering, Techniques and instrumentation

## Abstract

This paper presents a dual-band power amplifier (PA) using a meandered line bandstop filter (BSF). An important challenge addressed in this design is to achieve proper isolation between the operational bands of the amplifier. The proposed BSF provides isolation and efficiency, effectively separating the output power and power gain between the two operational bands. Additionally, a dual-mode bias circuit is designed to serve as an inductor choke and control the second harmonics for both operating frequencies simultaneously. Two dual-band PAs, utilizing LDMOS and GaN HEMT transistors, have been designed using the proposed output matching circuit, which incorporates the BSF, bias circuit, and compensation circuit. The results obtained from both PAs, employing different transistors, are identical. Based on the presented concepts, a dual-band PA with an LDMOS transistor has been fabricated and measured. The measurements reveal an efficiency of 79.23%, an output power of 39.85 dBm, and a power gain of 14.85 dB at a frequency of 0.7 GHz. Similarly, at a frequency of 1.9 GHz, the efficiency is 77.24%, the output power is 38.22 dBm, and the power gain is 13.22 dB.

## Introduction

**Figure 1:**
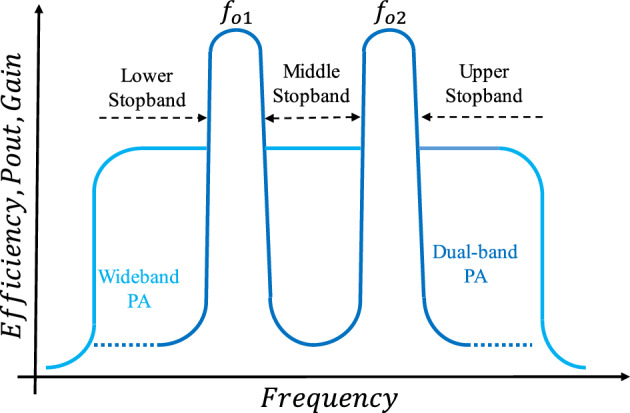
Efficiency, Pout and gain versus frequency for two modes of wide- and dual-bands.

Multistandard transceiver systems must handle different signals at standard-specified frequencies, meeting requirements in terms of signal quality and power efficiency. Wideband circuits are suitable for reconfigurable transceiver systems, but designing an output matching network for a wide operational bandwidth in order to achieve high efficiency and linearity is a challenging task. In^1^, a Class-J PA using a lumped π-type output matching network is presented to improve efficiency and linearity. The design of the output matching network is crucial, especially when the target bandwidth covers multiple separate communication standards, such as a wireless power transfer system for deep-implanted biomedical devices^2^. In this regard, an optimal solution is to use a matching circuit that operates only at specific standard frequencies. Multiband amplifiers are superior to their broadband counterparts because they do not suffer from in-band harmonic interference within the operating band. Therefore, there is no need to implement multiple circuits to control harmonic interference across the entire operational band. Multiband systems have gained popularity in recent years due to their compact size, low cost, and ease of implementation. Several dual-band PAs with different structures have been reported^3–16^. Challenges in dual-band PAs include efficiency, output power, gain, and cost, with the most significant challenge being the similarity of characteristics between the two bands. In the previous designs of dual-band amplifiers, the use of an element or circuit instead of an RF choke has not been thoroughly investigated. However, replacing RF chokes with stubs, which are integral to the amplifier circuit, can serve as effective RF chokes for both bands. Moreover, previous works have mainly focused on dividing the efficiency of the amplifiers into two bands, neglecting other factors. As shown in Fig. 1, wideband amplifiers exhibit improved output power, gain, and efficiency within a specific frequency range. On the other hand, in older dual-band PAs, only the efficiency is divided into two parts. In^15^, although the isolation of small-signal output power between the two operating bands is relatively suitable, the isolation of large-signal output power is weak. From this point of view, isolation between two bands should be created in terms of efficiency, gain and output power. The proper isolation level means the level less than 0 dBm, which in^15^, the output power between two bands has reached 10 dBm and does not have proper isolation for the output power. Consequently, if an unwanted signal with a frequency value between the two operating frequencies is applied to a dual-band amplifier, it will be amplified with suitable gain but low efficiency. In other words, dual-band amplifiers lack proper isolation between the two bands, particularly in the middle stopband. As depicted in Fig. 1, three stopbands are considered for a dual-band PA: lower, middle, and upper stopbands. Previous works have achieved suitable isolation in the upper and lower stopbands, as there is no amplification in these bands. However, in the middle band, only the efficiency decreases. These drawbacks can be observed in previous works^3–16^ where the output power and power gain are not isolated in the middle band. Isolation within the middle band is crucial for efficiency and linearity. Due to in-band harmonic interference, particularly in frequency bands below 1 GHz, unwanted harmonics are amplified^17–21^, leading to a reduction in linearity and efficiency. This issue becomes more pronounced in dual-band power amplifiers (PAs) where *f*_O2_ > 2*f*_O1_. Consequently, designers face a new challenge of designing a dual-band amplifier with mid-band isolation to optimize efficiency, gain, and output power.

In this paper, a band-stop filter (BSF) is employed to suppress all signals between the two operating bands with a high level of attenuation. The proposed BSF structure comprises spiral lines and utilizes the coupling effect to achieve a wide stopband. Additionally, a dual-mode bias circuit has been introduced to control the second harmonics. This circuit is utilized simultaneously for both operating bands, serving as a substitute for an RF choke.

The novelties of the proposed PA are summarized as follows:

- A novel harmonic control, which consist of BSF, bias and compensation circuit, is proposed and designed.
- A BSF with novel structure for dual-band is proposed in order to remove unwanted harmonics and also to create a very strong isolation between two operating bands for three parameters, i.e., efficiency, output power and power gain, whereas simultaneous isolation for all three parameters does not exist in previous published works. The proposed new structure of BSF consists of meandered lines and the coupling effect in order to reduce the overall size of the filter, to increase the number of transmission zeros and to extend stopband bandwidth.
- The proposed bias circuit is designed in such a way to act as RF chock for two specific frequencies, i,e., 0.7 and 1.9 GHz. Furthermore, the proposed bias circuit controls the second harmonics, i.e., 1.4 and 3.8 GHz, where this capability was not observed in the previous published works. The structure of the proposed bias circuit, consists of a quarter-wavelength line along with connected radial stubs to high impedance lines.
- A compensation circuit is proposed in order to match the harmonic control network with the transistor, so that two important transistors such as LDMOS and HEMT GaN can be used.

Consequently, the novel structure of the proposed harmonic control circuit in the PA provides proper results for dual-band applications, where the frequency gap between the two operating bands is relatively large.

## Proposed OMN design

The proposed dual-band PA is designed to operate at frequencies of 0.7 and 1.9 GHz. As mentioned earlier, the output matching network (OMN) of the amplifier consists of three main components: the band-stop filter (BSF), the bias circuit, and the compensation circuit.

### BSF design

As mentioned earlier, the isolation between the two bands is crucial for a dual-band PA. To achieve this, a band-stop filter (BSF) has been designed to suppress signals in the mid-band. Designing a BSF poses challenges such as achieving wide and deep stopbands, ensuring proper insertion and return losses in the lower and higher passbands, achieving a sharp roll-off, and maintaining a compact size. In the proposed design, these challenges have been taken into account.

**Figure 2:**
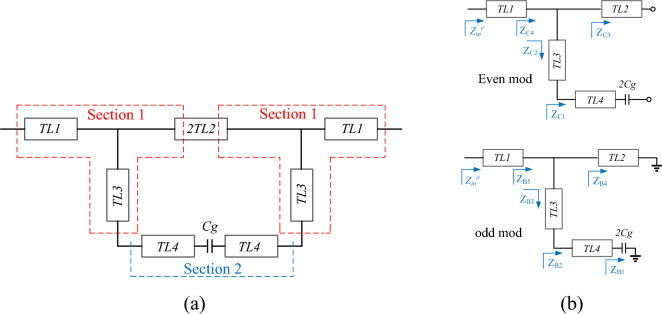
Proposed BSF, (**a**) equivalent circuit, (**b**) odd and even modes.

Figure 2 show equivalent circuit model of proposed BSF, and even/odd modes. The proposed equivalent circuit is based on the impedance and electrical length of the microstrip lines, and the coupling effect is equivalent to a *Cg* capacitor. In the design of the proposed BSF, high-impedance lines with an electrical length of ${90}^{o}$ ($\lambda /2$), low-impedance lines and coupling effect are used. The $\lambda /2$ lines are needed to create adequate transmission poles and sharp roll-off. The existence of the transmission poles at operational frequencies are necessary for the use of the proposed filter in an amplifier, in order to avoid the loss of the amplified power. The $\lambda /2$ lines, in addition to have transmission poles at the main frequency (*fo*_1_), creates alternating transmission zeros at frequencies of 3*f*_1_, 5*f*_1_, 7*f*_1_, etc. Low-impedance lines with coupling effect double the transmission zeros. That is, the coupling effect turns each transition zero into two, thereby increasing the stopband bandwidth. As shown in Fig. 2b, for analysis even and odd modes are employed as follows:

1 $${Z}_{B1}=\frac{1}{{2\pi f2C}_{g}},$$

2 $${Z}_{B2}={Z}_{4}\cdot \frac{{Z}_{B1}+j{Z}_{4}\mathrm{tan}{\theta }_{4}}{{Z}_{4}+j{Z}_{B1}\mathrm{tan}{\theta }_{4}},$$

3 $${Z}_{B3}=Z\cdot \frac{{Z}_{B2}+jZ\mathrm{tan}{\theta }_{3}}{Z+j{Z}_{B2}\mathrm{tan}{\theta }_{3}},$$

4 $${Z}_{B4}=jZ\mathrm{tan}{\theta }_{3},$$

5 $${Z}_{B5}=\frac{{Z}_{B4}\cdot {Z}_{B3}}{{Z}_{B4}+{Z}_{B3}},$$

6 $${Z}_{in}^{o}=Z.\frac{{Z}_{B5}+jZ\mathrm{tan}{\theta }_{1}}{Z+j{Z}_{B5}\mathrm{tan}{\theta }_{1}},$$

The step by step even mode input impedance is calculated:

7 $${Z}_{C1}=-j{Z}_{4}\mathrm{cot}{\theta }_{4},$$

8 $${Z}_{C2}=Z\cdot \frac{{Z}_{C1}+jZ\mathrm{tan}{\theta }_{3}}{Z+j{Z}_{C1}\mathrm{tan}{\theta }_{3}},$$

9 $${Z}_{C3}=-j\mathrm{Zcot}{\theta }_{2},$$

10 $${Z}_{C4}=\frac{{Z}_{C2}\cdot {Z}_{C3}}{{Z}_{C2}+{Z}_{C3}},$$

11 $${Z}_{in}^{e}={Z}_{C4}.\frac{Z+j{Z}_{C4}\mathrm{tan}{\theta }_{1}}{{Z}_{C4}+j\mathrm{Ztan}{\theta }_{1}},$$

since:

12 $${\mathrm{S}}_{21}=\frac{{Z}_{in}^{e}-{Z}_{in}^{o}}{({Z}_{in}^{e}+1)({Z}_{in}^{o}+1)}.$$

The condition for creation of transmission zero is S_21_ = 0, or ${Z}_{in}^{o}={Z}_{in}^{e}$^23^. In this design, firstly section 1 is designed and two transmission zeros have been created at 1.44 and 4.23 GHz. Then, according to the above analysis, the dimensions of the TL4 and the distance between them have been set so that two transmission zeros are placed at the frequencies of 1.077 and 1.38 GHz.

**Figure 3:**
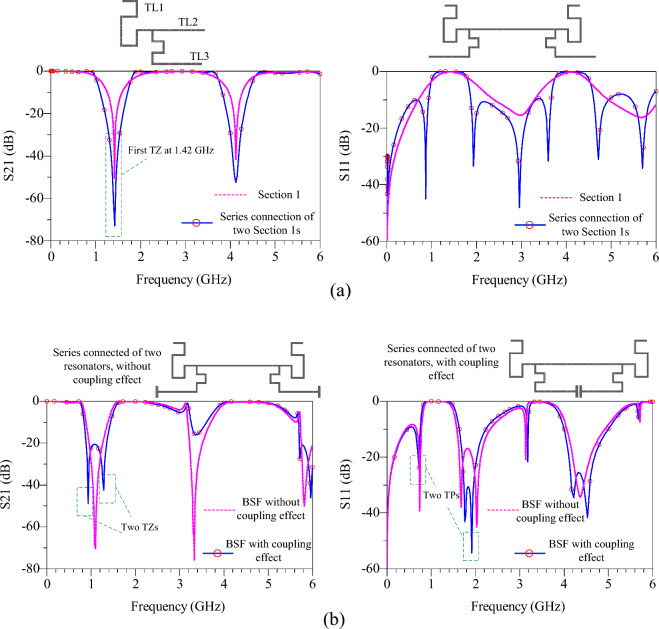
Design process of the proposed BSF, (**a**) layouts and simulation results of the section 1, series connection of the two section 1s, (**b**) layouts and simulation results of the proposed filter with and without coupling effect.

Figure 3 shows the design process of proposed BSF. According to Fig. 3a, the proposed filter has two sections: section 1 includes TL1, TL2 and TL3, which are $\lambda /2$ lines. Section 2 includes low-impedance of TL4 and the coupling effect between these two lines. In section 1, TL1 and TL2 do not play a role in changing the frequency of transmission zero, but they increase the roll-off and create transmission poles. The length of these lines is chosen as the length of the TL3 line (${\theta }_{1}={\theta }_{3}=2{\theta }_{2}={90}^{o}$). After design of section 1, by connecting two structures of section 1 in series, roll-off and suppression level are increased^22^. Next, by adding section 2, the number of transmission zeros in the previous structure are doubled, as shown in Fig. 3b. The transmission poles play a crucial role in minimizing insertion losses at the amplifier's operating frequencies. The design incorporates a safety margin to account for variations in the fabrication process and to reduce errors and defects. The safety margin is determined by the attenuation level of the transmission poles. As shown in Fig. 3b, the attenuation level of the first pole is − 22.5 dB at a frequency of 0.7 GHz, and the attenuation level of the second pole is − 44.5 dB at a frequency of 1.9 GHz. The safety margin is defined for S11 < − 15 dB, and it spans from 0.67 to 0.74 GHz for the first transmission pole and from 1.67 to 2.1 GHz for the second transmission pole. With the presence of these transmission poles, the insertion loss at the frequency of 0.7 GHz is measured to be 0.087, while at the frequency of 1.9 GHz, the insertion loss is 0.101. By achieving these specific insertion loss values, the proposed BSF successfully optimizes the performance of the power amplifier and ensures effective suppression of unwanted frequencies.

### The design process in the layout environment

After analyzing the equivalent circuit and obtaining the values, the design process is conducted in the layout environment. During this stage, electromagnetic (EM) simulations are performed using ADS software, and the results slightly differ from the schematic simulations. Therefore, the characteristics of the proposed filter lines are adjusted in the layout environment. Since the lengths of TL1 and TL3 are quite long, the overall dimensions of the band-stop filter (BSF) significantly increase. To address this issue, a spiral structure is employed for these two lines to reduce the size of the proposed filter. Based on the results of the proposed BSF, the insertion losses at the frequencies of 0.7 and 1.9 GHz have been reduced to less than 0.1 dB, owing to the presence of transmission poles. The stopband of the filter ranges from 0.89 to 1.35 GHz with a suppression level of 20 dB.

### Bias circuit

**Figure 4:**
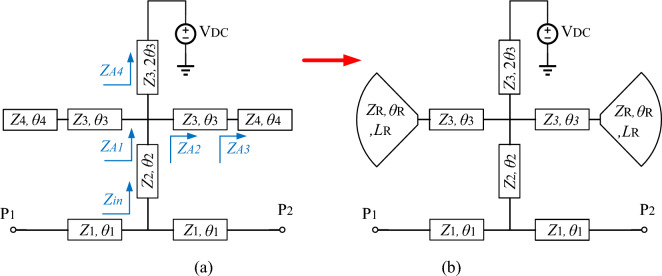
The equivalent model of proposed bias circuit, (**a**) without and, (**b**) with radial stub.

In power amplifiers, an RF-chock is used to prevent the AC signal leakage into the DC source. In microwave circuits, a quarter-wavelength line is used instead of RF-chock, which has low losses. However, this stub only blocks the main frequency and its odd harmonics. As result, the quarter-wavelength line cannot block the two frequencies in dual-band amplifiers. In this article, a dual-mode bias circuit is proposed which, in addition to blocking the two main frequencies, can also control the second harmonics of both main frequencies. The main characteristics of the proposed bias circuit include two transmission poles for the main frequencies of the amplifier and two transmission zeros for the second harmonics. Figure 4 shows the equivalent model of proposed bias circuit, based on the impedance and electrical length, with (a) and without (b) radial stubs. In this circuit, the TL1 lines are adjusted to control the imaginary part of the second harmonics according to the input/output impedance of the transistor. The TL2 line is a quarter-wavelength line to suppress the second signal (*f*_*O*2_). This line creates two goals of the proposed bias circuit, i.e., S_21_ = 1(@1.9 GHz), S_21_ = 0(@3.8 GHz). It should be noted that in the circuits of Fig. 4, for S_21_ = 1, the signal is completely transmitted from P1 to P2; And for S_21_ = 0, the signal is completely transmitted to the ground (second harmonic control). The other two goals, i.e., S_21_ = 1(@0.7 GHz), S_21_ = 0(@1.4 GHz), are achieved by TL3s and TL4s. To obtain the goals of S_21_ = 0 (@1.4 GHz) or S_11_ = 1(@1.4 GHz), firstly the input impedance ${Z}_{\mathrm{in}}$ is calculated as follows:

13 $${Z}_{A4}=j{Z}_{3}\mathrm{tan}2{\theta }_{3},$$

14 $${Z}_{A3}=-j{Z}_{4}\mathrm{cot}{\theta }_{4},$$

15 $${Z}_{A2}={Z}_{3}.\frac{{Z}_{A3}+j{Z}_{3}\mathrm{tan}{\theta }_{3}}{{Z}_{3}+j{Z}_{A3}\mathrm{tan}{\theta }_{3}} ,$$

16 $${Z}_{A1}=\frac{\frac{1}{2}{Z}_{A2}.{Z}_{A4}}{\frac{1}{2}{Z}_{A2}+{Z}_{A4}} ,$$

17 $${Z}_{\mathrm{in}}={Z}_{2}\cdot \frac{{Z}_{A1}+j{Z}_{2}\mathrm{tan}{\theta }_{2}}{{Z}_{2}+j{Z}_{A1}\mathrm{tan}{\theta }_{2}}.$$

**Figure 5:**
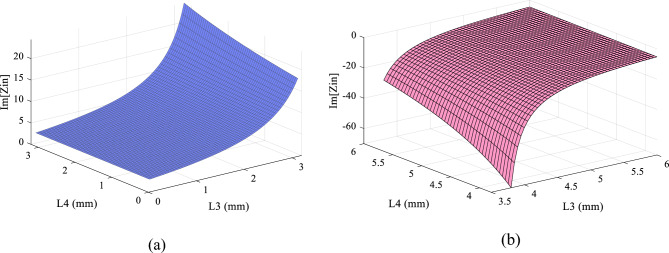
Im[Zin] versus L3 and L4 in ranges of (**a**) 0.1 to 3 mm and (**b**) 3.5 to 6 mm.

**Figure 6:**
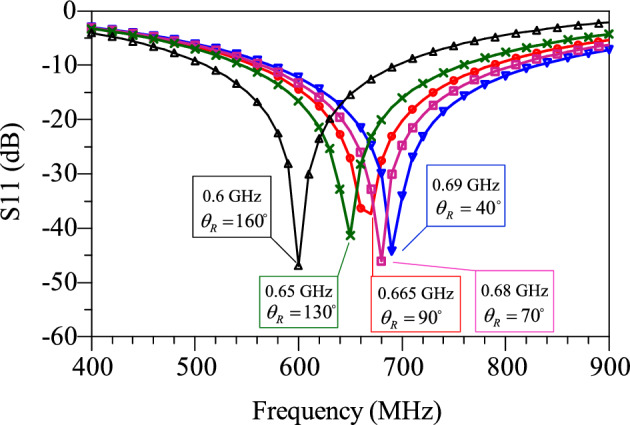
Location of 0.7 GHz transmission zero versus variation of ${\theta }_{R}$.

**Figure 7:**
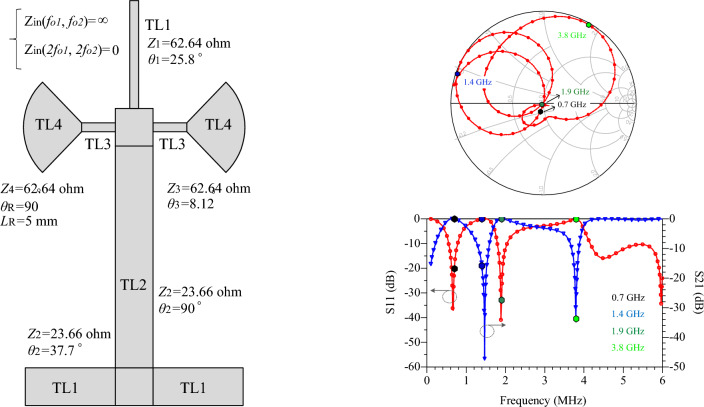
The layout of proposed bias circuit and its simulated response.

${Z}_{\mathrm{in}}$ is simplified by ${Z}_{2}=23 \mathrm{ohms}(\mathrm{low}-\mathrm{impedance}),$ ${\theta }_{2}=\frac{\uppi }{4}$, ${Z}_{3}={Z}_{4}=62.63 \mathrm{ohms}(\mathrm{high}-\mathrm{impedance})$ and ${\theta }_{2}=\frac{2\uppi f\sqrt{{\varepsilon }_{re}}}{C}.L$, which $L$ is the length of the lines, ${\varepsilon }_{re}$ is the effective dielectric constant of the substrate and $f$ is the frequency. The values of $62.63$ and $23 \mathrm{ohms}$ are the highest and lowest possible impedance of the microstrip line with substrate of RO4003. In order to reduce losses, the minimum width of microstrip lines is 0.8 mm, which is equivalent to $62.63$ ohms. Also, the maximum width is equivalent to 23 ohms. The value of ${Z}_{2}, {Z}_{3} \mathrm{and} {Z}_{4}$ has been obtained according to the employed substrate of RO4003 (${\varepsilon }_{re}=3.38, \mathrm{tan}D=0.0022 \mathrm{and thickness}=20 \mathrm{mil}$). As a result, ${Z}_{\mathrm{in}}$ depends on two parameters L3 and L4. The goal of S_11_ = 1(@1.4 GHz) or S_11_ = − ∞ dB (@1.4 GHz) means creating a transmission pole at GHz. As a result, ${Z}_{\mathrm{in}}$ depends on two parameters L3 and L4. The goal of S_11_ = 1(@1.4 GHz) or S_11_ = − ∞ dB (@1.4 GHz) means creating a transmission pole at 1.4 GHz. As a result, Re [${Z}_{\mathrm{in}}$] = 50 and Im [${Z}_{\mathrm{in}}$] = 0. At frequency of 1.4 GHz, Im[${Z}_{\mathrm{in}}$] versus of L3 and L4 are shown in Fig. 5a,b*,* respectively. The value of L3 and L4 is chosen so that ${Z}_{\mathrm{in}}$ has the minimum impedance at the frequency of 1.4 GHz. The goal of S_21_ = 1(@0.7 GHz) means creating a transmission pole between two ports P1 and P2. TL3 and TL4 lines create this transmission pole. Since the characteristics of the TL4 line depends on the input impedance calculated in the previous section, a radial stub is used instead of the TL4 to set this transmission to zero. The radial stub has a new feature ${\theta }_{R}$ that is used to adjust the position of the transmission zero. Figure 6 shows the variation of transmission zero at 0.7 GHz versus ${\theta }_{R}$ variation. Finally, based on the aforementioned analysis, the characteristics of the dual-mode bias circuit lines are obtained. Figure 7 shows the proposed bias circuit along with its simulation response in the post layout section. The dual-mode bias circuit is considered in a dual-band PA, for both V_GG_ and V_DD_ biases. The advantage of the proposed bias circuit from broadband matching network is that the proposed circuit, using a new structure, controls the harmonics only for the two operating frequencies of the amplifier with more accuracy and attenuation level.

The simulation results for both power amplifiers (PAs) based on the proposed output matching network (OMN) with different transistors are presented in Table 1. It is evident from the table that by optimizing only the compensator part of the proposed OMN, satisfactory results have been achieved for the two conventional transistors in PA design. The lower gain and output power observed at the frequency of 1.9 GHz for the LDMOS transistor can be attributed to one of its characteristics at this frequency. However, the simulation results indicate that when using the CGH400010F transistor, the power gain, output power, and efficiency are comparable in both operating bands.

**Table 1:** Performance comparison the proposed PA with two different transistors.

Transistor	Pout (dBm)	Gain (dB)	DE (%)	V_DD_ (V)
AFT27S006N	40.7/38.58	15.7/13.58	81.56/79.4	25
CGH400010F	39.2/39.75	14.2/14.75	79.23/80.7	28

## Design of proposed dual-band PA

**Figure 8:**
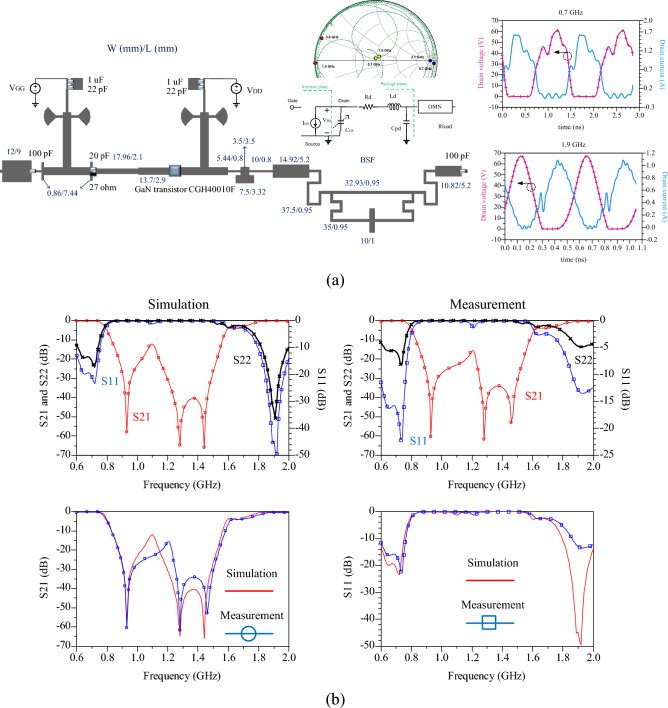
(**a**) The schematic of the proposed dual-band PA, input impedance at intrinsic plane and drain voltage/current waveforms, (**b**) simulated and measured s-parameters from transistor output to the PA output.

The schematic of the proposed power amplifier (PA) is depicted in Fig. 8a. The transistor is biased using two dual-mode bias circuits. The stability circuit comprises a 27-Ω resistor and a 20-pF capacitor (GQM1875C). The DC block capacitors are 100 pF (GQM2195C Murata), the bypass capacitors are 12 pF (GRM1885C Murata), and the decoupling capacitors are 1 µF. The input matching circuit is designed while considering the bias circuit. The compensator circuit consists of three microstrip lines, which help reduce the insertion loss at 1.9 GHz by setting the transmission pole. In the layout of the PA shown in Fig. 8, a compensation circuit consisting of four lines is placed after the bias circuit. These lines serve to match the output matching circuit to the transistor's output impedance and also play a role in controlling the third harmonics. The specifications of the compensator lines are determined through an optimization process in the software after selecting the transistor. Finally, the proposed band-stop filter (BSF) is implemented. The voltage values of V_DD_ = 25 V and V_GG_ = 2.3 V are selected for the 6-W NXP AFT27S006N LDMOS transistor. The input impedance of the proposed matching circuit, which includes the bias circuit, compensation circuits, and BSF, at the I-gen plane of the transistor, is illustrated in Fig. 8a. According to this figure, at the frequencies of 0.7 GHz and 1.9 GHz, the first harmonics exhibit a good match (50 ohms), the impedance of the second harmonics is close to zero due to the role of the proposed bias circuit and BSF, and the impedance of the third harmonics at the I-gen plane is close to infinity due to the compensation circuit of the transistor. Additionally, the current and drain voltage waveforms at the I-gen plane demonstrate that the third harmonics' impedance is close to infinity due to the compensation circuit, as seen in Fig. 8a. The current waveform is semi-sinusoidal, and the voltage waveform is close to square, indicating effective control of the second and third harmonics. The simulated and measured insertion loss from the transistor output to the PA output is illustrated in Fig. 8b. The results include S_21_, S_11_, and S_22_ responses in two states: Simulation and measurement. According to the results, the simulation and measurement insertion loss values at 0.7 GHz are − 0.13 dB and − 0.19 dB, respectively. Also, the simulation and measurement insertion loss values at 1.9 GHz are − 0.18 dB and − 0.315 dB, respectively.

**Figure 9:**
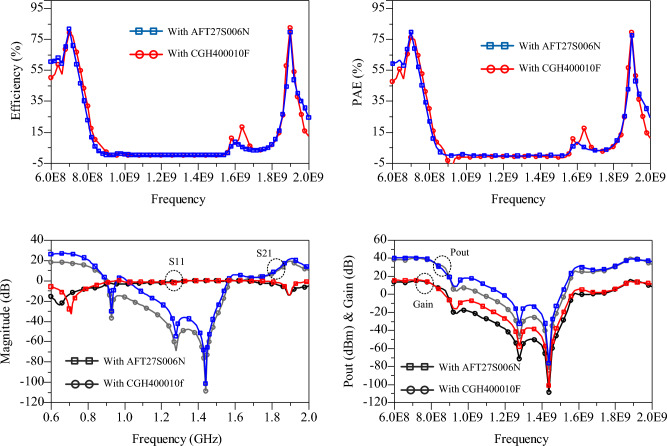
Simulation results of the proposed PA with two transistors of AFT27S006N and CGH400010F.

Recently, there has been a special interest in using GaN HEMT transistors for power amplifier (PA) design, as they offer high efficiency and output power. However, these transistors also come with increased fabrication costs. On the other hand, the utilization of low-cost LDMOS transistors can be a suitable option for applications below 3 GHz. The proposed design is significant in this regard, as by optimizing the compensation circuit section, it can accommodate any transistor. Hence, the proposed design has been tested using two transistors: LDMOS (AFT27S006N) and GaN HEMT (CGH40010F). The simulation results for both amplifiers based on these two different types of transistors are shown in Fig. 9. It can be observed that the difference between the two simulated results is minimal, and both amplifiers exhibit excellent efficiency and gain in the two bands. Furthermore, the beneficial role of the proposed BSF in achieving a high level of isolation between the two bands is clearly evident in Fig. 9.

## Fabrication and measurement process

**Figure 10:**
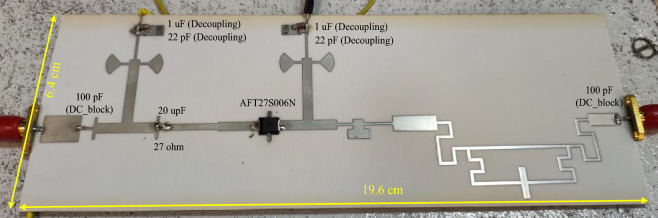
Photograph of the fabricated dual-band PA.

**Figure 11:**
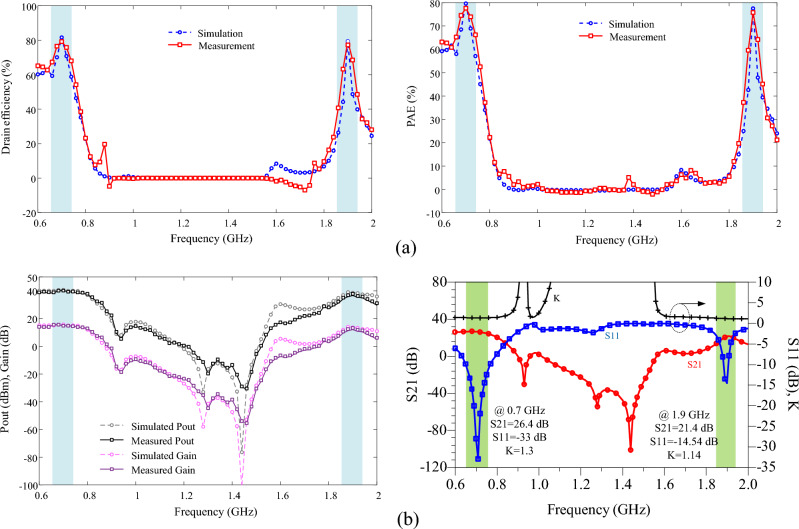
Simulated and measured results of proposed dual-band PA, (**a**) DE and PAE versus frequency, (**b**) S-parameters, Pout and gain versus frequency.

**Figure 12:**
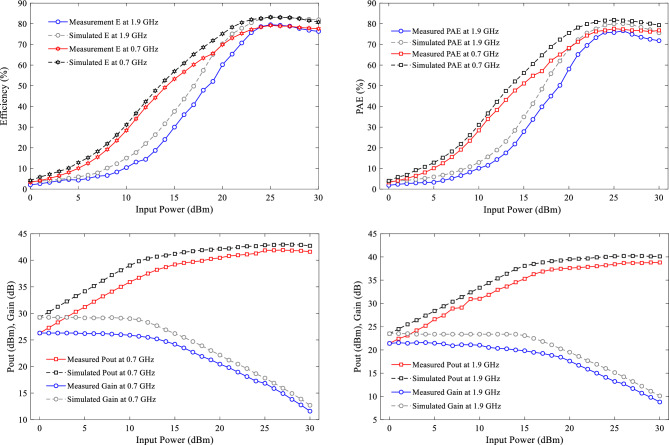
Simulation and measurement of drain efficiency, PAE, Pout and Gain versus of input power at 0.7 and 1.9 GHz.

In order to confirm the proposed design, an PA using the LDMOS AFT27S006N transistor and the mentioned elements are fabricated on the substrate of RO4003 (${\varepsilon }_{re}=3.38, \mathrm{tan}D=0.0022 \mathrm{and thickness}=20 \mathrm{mil}$). As mentioned earlier, the output matching network has demonstrated good performance for the two transistors, AFT27S006N and CGH400010F. Therefore, the LDMOS-based design has been chosen to reduce costs in the fabrication process. The large-signal performance of the proposed power amplifier (PA) is measured using an R&S SMA100B RF and Microwave Analog Signal Generator and an R&S HMS3010 Spectrum Analyzer. The photograph of the fabricated PA is shown in Fig. 10. The simulation and measurement results, which are in good agreement, are illustrated in Fig. 11. The measured results indicate that at a frequency of 0.7 GHz (first operating band), the output power (Pout) is 39.85 dBm, the power gain is 14.85 dB, and the power-added efficiency (PAE) at 10 dB gain compression is 77.6%. Similarly, at a frequency of 1.9 GHz (second operating band), the measured Pout is 38.22 dBm, the measured gain is 13.22 dB, and the measured PAE is 75.76%. The measured drain efficiencies at 10 dB gain compression are 79.23% and 77.24% at 0.7 GHz and 1.9 GHz, respectively. Also, the drain efficiency values at 3dB gain compression for frequencies of 0.7 and 1.9 GHz are equal to 76.3% and 74.9%, respectively. It is worth noting that each operating band has a very narrow bandwidth. For a frequency of 0.7 GHz, the bandwidth ranges from 0.67 to 0.73 GHz with an efficiency of 70%. Similarly, for a frequency of 1.9 GHz, the bandwidth ranges from 1.89 to 1.91 GHz with an efficiency of 67%. One of the main advantages of the proposed design is demonstrated in the large-signal results. The isolation between the two bands is observed from 0.9 to 1.76 GHz, within which the efficiency is less than 18%, the output power is less than 24.8 dBm, and the power gain is less than 0 dB. Additionally, the proposed band-stop filter (BSF) effectively suppresses the power gain and output power between the two operational bands of the amplifier with a high level of suppression. The results of the small-signal performance of the proposed PA are illustrated in Fig. 11b. At the operating frequencies of 0.7 GHz and 1.9 GHz, the return losses are 30 dB and 14.54 dB, and the measured S_21_ values are 26.3 dB and 21.39 dB, respectively. Figure 12 shows the simulated and measured drain efficiency, PAE, Pout, and gain versus input power (Pin) at the two operating frequencies of 0.7 GHz and 1.9 GHz. The output power changes from 0 to 30 dBm, which is measured for each value of input power, output power, gain and efficiency. According to the simulation and measurement results, there is a slight difference between the simulation and measurement results, so that the measurement results were slightly weaker than the simulation results. The difference between the measurement and simulation results is due to fabrication and measurement problems, including losses in connections and connectors SMA, as well as tolerance and parasitic effects of lumped elements. Nevertheless, the values of output power, power gain and output efficiency of the amplifier have been very suitable in two operating bands.

### Linearity tests

**Figure 13:**
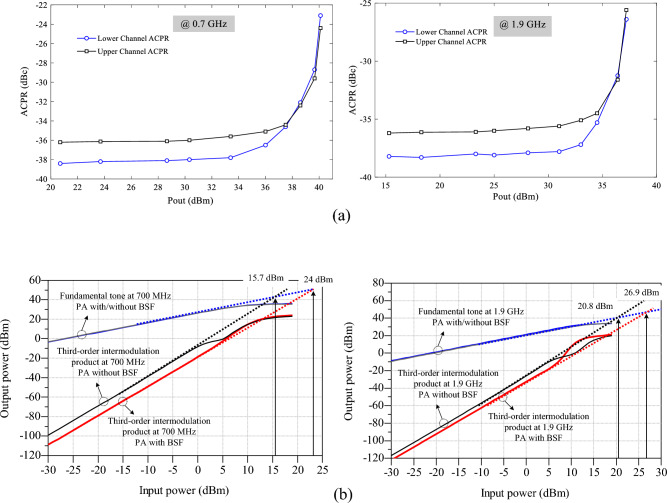
Linearity results of the proposed amplifier (**a**) ACPR and (**b**) IIP3 at 0.7 and 1.9 GHz.

To evaluate the linearity of the power amplifier (PA) in modern wireless communication systems, the adjacent channel power ratio (ACPR) is simulated. The simulation is conducted with a 5-MHz Wideband Code Division Multiple Access @ ± 2 MHz offset and a peak-to-average power ratio (PAPR) of 6.5 dB at frequencies 0.7 and 1.9 GHz, as illustrated in Fig. 13a. The ACPR values at both frequencies are observed to be lower than − 22 dBc, indicating excellent linearity performance. Furthermore, the linearity results in terms of the third-order input intercept point (IIP3) are simulated for the proposed PA, considering two modes: with and without the band-stop filter (BSF), as depicted in Fig. 13b. It can be observed from the figure that the presence of the proposed BSF improves the linearity parameter (IIP3) for both operating bands. The strong isolation between the two bands provided by the BSF suppresses harmonics, particularly the third harmonic, resulting in enhanced linearity for the first band of the dual-band PAs. Additionally, the compensator circuit effectively suppresses the third-order harmonic in the second band.

The measured results demonstrate the satisfactory performance of the proposed power amplifier (PA). To compare its performance with previous works, Table 2 presents a fair comparison. Based on the table, the proposed PA exhibits significant superiority in terms of efficiency, output power, and mid-band isolation compared to other works.

**Table 2:** Performance comparison with other dual-band PAs (a: at 3 dB gain compression, b: PAE values, DB: dual-band, HCN: harmonic control circuit).

Ref	Process/VDD (V)	*f*_1_/*f*_2_ (GHz)	DE (%) ^a^	Pout (dBm)	Gain (dB)	Pout Mid-band level (dBm) (BW in GHz)	Gain Mid-band level (dB) (BW in GHz)	Design method
^3^	GaN HEMT/28	1/2.3	72.474.1	41.6/42.1	11.6/11.2	40 (1.2–1.8)	11 (1.2–1.8)	DB coupler
^5^	GaN HEMT/28	0.7/1.75	72.5/70.5 ^b^	40.5/41	16/13	No isolation	No isolation	Dynamical continuous-mode criterions
^6^	GaN HEMT/28	2.6/3.5	71/64	44.6/43.5	–	NA	NA	DB impedance transformer and reactance compensation network
^9^	GaN HEMT/24	2.45/5.76	62.9/61.7	39/35	11/7	–	NA	distributed element-based load network
^10^	GaN HEMT/28	1.62/2.08	71.5/73 ^b^	40.2/40	–	NA	NA	DB harmonic-tuned
^11^	GaN HEMT/28	1.72/2.14	74.9/75.5 ^b^	40.5/40.9	–	38 (1.8–2.1)	12 (1.8–2.1)	DB-HCN and compensation circuit
^13^	0.1-um GaAS/7	6/16	55/53	26/25.5	14.9/9	22 (8–15)	NA	harmonic terminations
^14^	GaN HEMT/28	2.6/3.5	76.7/72.8	42.4/41.1	11.5/10.5	38 (2.7–3.4)	NA	harmonic turning and RTF
^15^	GaN HEMT/28	1.8/2.4	64/54	43/43	10/10	35 (2–2.2)	8 (2–2.2)	Concurrent DB Doherty
^16^	GaN HEMT/28	2.45/3.3	53/46	33/32.5	10/9	20 (2.7–2.9)	− 4 (2.7–2.9)	Concurrent DB harmonic tuned
This work	LDMOS/25	0.7/1.9	76.3/74.9	39.85/38.22	14.85/13.22	0 (1–1.5)	0 (0.86–1.5)	BSF, bias and compensator circuits

## Conclusion

In this paper, strong isolation between two operational bands is achieved in a dual-band power amplifier (PA) by utilizing a meandered line bandstop filter (BSF). A dual-mode bias circuit, based on radial stubs, has been designed to resonate (in an open circuit condition) at the first harmonics and control (in a short-circuit condition) the second harmonics. By incorporating the proposed BSF, bias circuit, and adjusting the compensator circuit, the dual-band PA is designed using both LDMOS and GaN HEMT transistors. The results obtained from both designs are identical, indicating that this design method is applicable to any transistor. Finally, a dual-band PA based on the LDMOS transistor is fabricated and measured using the aforementioned techniques. The measurement results demonstrate significant advantages of the proposed PA, including efficiency, output power, power gain, isolation between the two bands, and linearity. This design method holds promise and potential for the development of multi-band communication system such as wireless power transfer system for deep-implanted biomedical devices.

## Data Availability

The calculated results during the current study are available from the corresponding author on reasonable request.
